# Association Between Cataract Extraction and Development of Dementia

**DOI:** 10.1001/jamainternmed.2021.6990

**Published:** 2021-12-06

**Authors:** Cecilia S. Lee, Laura E. Gibbons, Aaron Y. Lee, Ryan T. Yanagihara, Marian S. Blazes, Michael L. Lee, Susan M. McCurry, James D. Bowen, Wayne C. McCormick, Paul K. Crane, Eric B. Larson

**Affiliations:** 1 Department of Ophthalmology, University of Washington, Seattle; 2 Roger and Angie Karalis Johnson Retina Center, Seattle, Washington; 3 Department of General Internal Medicine, University of Washington, Seattle; 4 School of Nursing, University of Washington, Seattle; 5 Department of Neurology, Swedish Medical Center, Seattle, Washington; 6 Kaiser Permanente Washington Health Research Institute, Seattle, Washington

## Abstract

**Importance:**

Visual function is important for older adults. Interventions to preserve vision, such as cataract extraction, may modify dementia risk.

**Objective:**

To determine whether cataract extraction is associated with reduced risk of dementia among older adults.

**Design, Setting, and Participants:**

This prospective, longitudinal cohort study analyzed data from the Adult Changes in Thought study, an ongoing, population-based cohort of randomly selected, cognitively normal members of Kaiser Permanente Washington. Study participants were 65 years of age or older and dementia free at enrollment and were followed up biennially until incident dementia (all-cause, Alzheimer disease, or Alzheimer disease and related dementia). Only participants who had a diagnosis of cataract or glaucoma before enrollment or during follow-up were included in the analyses (ie, a total of 3038 participants). Data used in the analyses were collected from 1994 through September 30, 2018, and all data were analyzed from April 6, 2019, to September 15, 2021.

**Exposures:**

The primary exposure of interest was cataract extraction. Data on diagnosis of cataract or glaucoma and exposure to surgery were extracted from electronic medical records. Extensive lists of dementia-related risk factors and health-related variables were obtained from study visit data and electronic medical records.

**Main Outcomes and Measures:**

The primary outcome was dementia as defined by *Diagnostic and Statistical Manual of Mental Disorders* (Fourth Edition) criteria. Multivariate Cox proportional hazards regression analyses were conducted with the primary outcome. To address potential healthy patient bias, weighted marginal structural models incorporating the probability of surgery were used and the association of dementia with glaucoma surgery, which does not restore vision, was evaluated.

**Results:**

In total, 3038 participants were included (mean [SD] age at first cataract diagnosis, 74.4 (6.2) years; 1800 women (59%) and 1238 men (41%); and 2752 (91%) self-reported White race). Based on 23 554 person-years of follow-up, cataract extraction was associated with significantly reduced risk (hazard ratio, 0.71; 95% CI, 0.62-0.83; *P* < .001) of dementia compared with participants without surgery after controlling for years of education, self-reported White race, and smoking history and stratifying by apolipoprotein E genotype, sex, and age group at cataract diagnosis. Similar results were obtained in marginal structural models after adjusting for an extensive list of potential confounders. Glaucoma surgery did not have a significant association with dementia risk (hazard ratio, 1.08; 95% CI, 0.75-1.56; *P* = .68). Similar results were found with the development of Alzheimer disease dementia.

**Conclusions and Relevance:**

This cohort study found that cataract extraction was significantly associated with lower risk of dementia development. If validated in future studies, cataract surgery may have clinical relevance in older adults at risk of developing dementia.

## Key Points

**Question:**

Is cataract extraction associated with reduced risk of developing dementia?

**Findings:**

In this cohort study assessing 3038 adults 65 years of age or older with cataract enrolled in the Adult Changes in Thought study, participants who underwent cataract extraction had lower risk of developing dementia than those who did not have cataract surgery after controlling for numerous additional risks. In comparison, risk of dementia did not differ between participants who did or did not undergo glaucoma surgery, which does not restore vision.

**Meaning:**

This study suggests that cataract extraction is associated with lower risk of developing dementia among older adults.

## Teaser

This cohort study analyzing data obtained from the Adult Changes in Thought study assesses whether cataract extraction is associated with reduced risk of dementia among adults 65 years of age or older.

## Introduction

Dementia affects nearly 50 million people worldwide, and no effective treatments exist.^1^ Efforts to reduce risk or delay dementia onset are increasingly important, as noted in the recent 2020 *Lancet* Commission report.^1^ Twenty percent of adults older than 65 years in the United States experience significant sensory impairment, such as vision or hearing loss, even with correction.^2^ Addressing sensory loss that affects a substantial portion of older adults may be a potentially modifiable risk factor for dementia in late life.^1,3^ Because sensory impairments and dementia are both strongly associated with aging,^4^ more knowledge about the association between sensory impairment and dementia may have important implications for individual and global public health, particularly if interventions to improve sensory function reduce dementia risk.

Visual impairment is an important dementia risk.^5,6^ Cataract is a leading cause of blindness worldwide, affecting more than 35 million people globally and causing blindness in approximately 20 million.^7^ Cataract affects most older adults at risk of dementia. However, there are conflicting results regarding the association between cataract extraction and cognitive impairment or dementia.^8,9,10^

We hypothesized that older adults with cataract who undergo cataract extraction may have a lower risk of developing dementia compared with participants who do not undergo cataract surgery or participants who undergo other eye procedures that do not restore vision, such as glaucoma surgery. Previous studies exploring this association have been limited by small sample sizes, cross-sectional designs, and varying qualities of dementia assessment.^11,12^ More importantly, these studies have failed to account for healthy patient bias (ie, when surgery is more likely in healthier individuals with the same cataract severity).

To the best of our knowledge, no study has compared associations between cataract extraction and dementia with other ophthalmic surgical procedures. To address the potential of healthy patient bias, we included glaucoma surgery in our analyses. We used extensive data from the Adult Changes in Thought (ACT) study to address these questions. We examined whether cataract extraction was associated with a lower risk of dementia, and we used the same modeling approach to examine whether glaucoma surgery was associated with a lower risk of dementia.

## Methods

### Study Design and Setting

Detailed study methods have been published.^13,14^ In brief, the ACT study began during the period from 1994 to 1996 and is an ongoing, population-based, prospective cohort study of older adults who are randomly selected and recruited from Kaiser Permanente Washington membership rolls and then followed up until the development of dementia.^13^ At enrollment and during biennial visits, participants receive standardized cognitive screening tests, brief physical evaluations, and medical history and risk factor assessments.^14,15^ This study was approved by the institutional review boards of Kaiser Permanente Washington and the University of Washington and was conducted in accordance with the Declaration of Helsinki. All participants provided written informed consent. No one received compensation or was offered any incentive for participating in this study.

### Participants

Participants were 65 years of age or older and dementia free at enrollment. In this cohort study, we included all ACT study participants who had received a diagnosis of cataract before onset of dementia and had at least 1 study visit after cataract diagnosis (eMethods 1 in the Supplement). For the glaucoma sensitivity analyses, we included participants who received a diagnosis of glaucoma and had follow-up data before the onset of dementia.

### Variables, Measurement, and Data Sources

The primary exposure of interest was cataract extraction as a time-varying covariate.^16,17,18,19,20^ Participants are evaluated biennially with the Cognitive Abilities Screening Instrument (CASI), which ranges from 0 to 100, with higher scores indicating better abilities.^21^ Participants with CASI scores of 85 or lower undergo a standardized diagnostic evaluation, including physical and neurologic examinations, an extensive medical record review, and a battery of neuropsychological tests^22^ (additional details in eMethods 2 in the Supplement). Our primary outcome was all-cause dementia defined by the *Diagnostic and Statistical Manual of Mental Disorders* (Fourth Edition),^23^ and our secondary outcome included probable or possible Alzheimer disease (AD) dementia diagnosed at a multidisciplinary consensus conference using the National Institute of Neurological and Communicative Disorders and Stroke and the Alzheimer’s Disease and Related Disorders Association criteria.^24,25^

The following variables were based on self-reported medical history at enrollment and were updated at each biennial follow-up: smoking, hypertension, congestive heart failure, diabetes, history of cardiovascular disease (myocardial infarction, angina, coronary artery bypass grafting, or angioplasty), and cerebrovascular disease (stroke, transient ischemic attack, or carotid endarterectomy). Participants were asked at each follow-up whether they had any difficulty with distance or near vision, even with corrective lenses. The health care utilization rate was assessed as the number of ambulatory visits per year in the 5 years prior to cataract diagnosis. For individuals with less than 5 years of electronic health records before cataract diagnosis, the rate was calculated based on the number of days enrolled at Kaiser Permanente Washington. Data on ophthalmic diagnoses and procedures, such as cataract, glaucoma, and related surgical procedures, were extracted from participants’ electronic medical records, available from 1993 onward (1 year prior to the initial enrollment period of the ACT study) (eMethods 3, eTable 1 in the Supplement).

### Statistical Analysis

We used Cox proportional hazards regression models with age as the time axis. All surgical procedures were treated as time-varying variables. Censoring occurred at death, dropout, or last visit. We also evaluated recent (surgery within 0-5 years) vs long-term (>5 years) cataract surgery associations with dementia risk. Model assumptions were assessed and found to be tenable.

All models were adjusted for years of education, self-reported White race, and smoking history and stratified by any apolipoprotein E ε4 (*APOE* ε4) alleles, sex, and age groups at cataract diagnosis (<68, 68-71, 72-76, and ≥77 years, which approximately correspond to quartiles of age at cataract diagnosis in our data). The following potential confounders of overall health were adjusted for in expanded models: diabetes, systolic blood pressure, hypertension, heart disease, cardiovascular disease, body mass index, self-rated health, Charlson Comorbidity Index,^26^ number of activities of daily living limitations, at least 15 minutes of physical activities 3 times per week, performance-based physical function scores, Center for Epidemiologic Studies Depression Scale scores,^27^ retirement status, and self-reported difficulty with distance or near vision. Data used in the analyses were collected from 1994 through September 30, 2018, and all data were analyzed between April 6, 2019, and September 15, 2021.

For model 1, we performed several sensitivity analyses: (1) excluding the 1994-1996 enrollment cohort, (2) counting the first 2 years after cataract surgery as unexposed person-time for development of dementia, (3) adjustment for health-related confounders already mentioned, (4) including only participants with incident cataract diagnosis after enrollment in the ACT study, (5) adjusting the incident model for most recent CASI score at the time of cataract diagnosis and varying the 5-year threshold for recent vs long-term associations to (6) a 2-year window and (7) a 10-year window. For model 2, we used marginal structural models to address healthy patient bias.^28^ Details of the models are given in eMethods 4 in the Supplement. For model 3, we repeated our primary model with glaucoma surgery as the exposure among people with glaucoma. All statistical analyses were conducted using Stata, version 17.0 (StataCorp LLC) and SAS, version 9.4 (SAS Institute Inc). A 2-sided *P* < .05 was considered statistically significant.

## Results

### Study Participants, Descriptive Data, Cataract Diagnosis, and Surgery Outcomes

**Figure 1.**
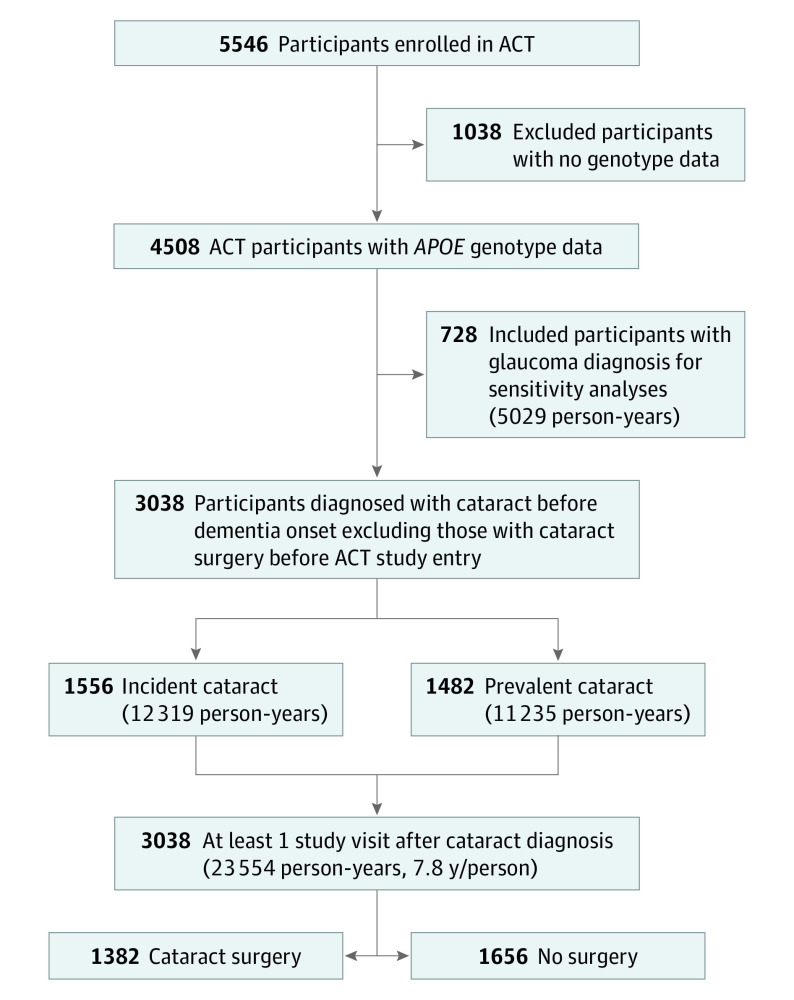
Flow Diagram of Study Population Inclusion ACT indicates Adult Changes in Thought; *APOE*, apolipoprotein E.

Of 5546 ACT participants, 4508 had *APOE* genotype data. Of these participants, 3038 (67% of participants who had *APOE* genotype data) received a diagnosis of a cataract before dementia onset or the end of the study, did not have surgery before the ACT study baseline, and had 1 or more study visits after cataract diagnosis (Figure 1). The mean (SD) age of the 3038 participants at first cataract diagnosis was 74.4 (6.2) years, 1800 participants (59%) were women, 1238 participants (41%) were men, and 2752 (91%) were self-reported White race. During the follow-up of 23 554 person-years (mean [SD] follow-up of 7.8 [5.1] years/person), there were 853 cases of incident dementia and 709 cases of incident AD dementia. Approximately one-half of the participants (n = 1382 [46%]) underwent cataract extraction (Table 1). Additional information on the study cohort are shown in eResults 1 and 2 and eTable 5 in the Supplement.

**Table 1.** Demographic and Health Characteristics by Subsequent Surgery Status^a^

Characteristic	No. (%) of participants			*P* value^b^
	Overall (n = 3038)	Cataract surgery (n = 1382)	No surgery (n = 1656)	
Female	1800 (59)	866 (63)	934 (56)	<.001
Male	1238 (41)	516 (37)	722 (44)	
Age at ACT study entry, mean (SD), y	73.8 (6.0)	73.3 (5.6)	74.2 (6.3)	<.001
Age at first cataract diagnosis, mean (SD), y	74.4 (6.2)	74.0 (5.9)	74.8 (6.5)	<.001
Years of education, mean (SD), y	14.8 (3.2)	14.8 (3.1)	14.7 (3.2)	.33
Self-reported White race	2752 (91)	1250 (90)	1502 (91)	.81
Any *APOE* ε4 alleles	803 (26)	354 (26)	449 (27)	.35
Past or current smoker	1559 (51)	726 (53)	833 (50)	.22
BMI, mean (SD)	27.4 (4.9)	27.3 (4.9)	27.5 (4.8)	.13
Ever reported having diabetes	325 (11)	146 (11)	179 (11)	.80
Ever reported having hypertension	1326 (45)	590 (43)	736 (45)	.29
Prevalent MI, angina, CABG, or angioplasty	539 (18)	241 (17)	298 (18)	.65
Ever reported stroke, TIA, or CEA	277 (9)	111 (8)	166 (10)	.06
CES-D Scale score, mean (SD)	3.6 (4.1)	3.5 (4.0)	3.6 (4.2)	.92
Systolic blood pressure, mean (SD), mm Hg	139.0 (20.3)	137.3 (19.8)	140.4 (20.6)	<.001
Poor or fair self-rated health	417 (14)	164 (12)	253 (15)	.006
Any ADL impairment	663 (22)	288 (21)	375 (23)	.23
No. of ADL impairments, mean (SD)	0.3 (0.8)	0.3 (0.7)	0.4 (0.8)	.15
At least 15 min of activity 3 times/wk	2139 (71)	983 (71)	1156 (70)	.48

Abbreviations: ACT, Adult Changes in Thought; ADL, activity of daily living; *APOE* ε4, apolipoprotein E ε4; BMI, body mass index (calculated as weight in kilograms divided by height in meters squared); CABG, coronary artery bypass grafting; CEA, carotid endarterectomy; CES-D, Center for Epidemiologic Studies Depression; MI, myocardial infarction; TIA, transient ischemic attack.

^a^ Demographic and health characteristics at first cataract diagnosis or at ACT study entry for those with prevalent cataract.

^b^ The χ^2^ test for dichotomous variables and the Wilcoxon rank sum test for continuous variables.

### All-Cause Dementia Risks

**Figure 2.**
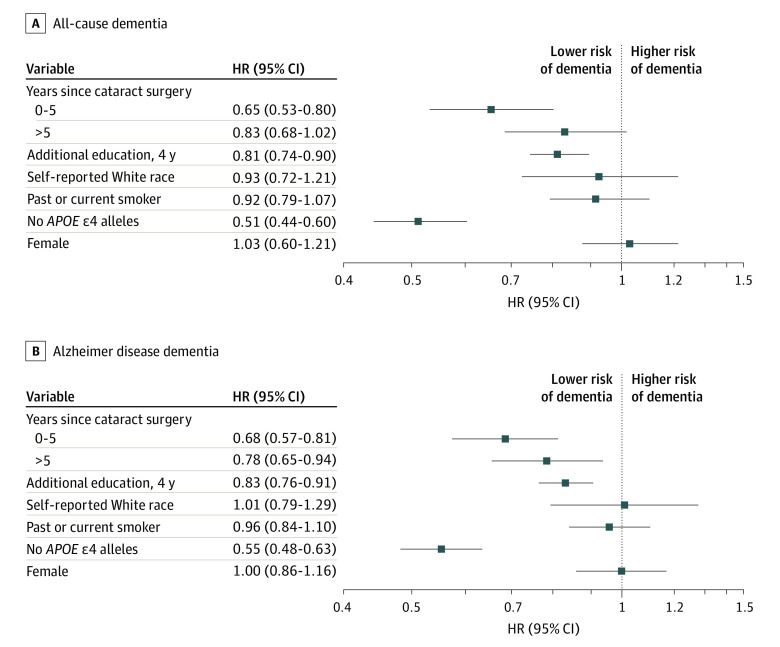
Risks of Developing All-Cause Dementia and Alzheimer Disease Dementia Hazard ratios (HRs) and 95% CIs for the development of all-cause dementia as defined by the *Diagnostic and Statistical Manual of Mental Disorders* (Fourth Edition) (A) and the development of probable or possible Alzheimer disease dementia as defined by the National Institute of Neurological and Communicative Disorders and Stroke and the Alzheimer’s Disease and Related Disorders Association criteria (B). In model 1, apolipoprotein E (*APOE*) genotype and sex were stratifying variables to meet proportional hazards assumptions, as was a categorical variable for age at first cataract diagnosis; however, for this illustration, *APOE* genotype and sex are included as covariates so that their associations can be compared. Age was the time axis.

Cataract extraction was significantly associated with a lower adjusted hazard ratio (HR) for dementia (HR, 0.71; 95% CI, 0.62-0.83; *P* < .001) (Table 2). This finding of lower risk was stronger during the first 5 years following cataract surgery (HR, 0.68; 95% CI, 0.56-0.81; *P* < .001) compared with later years (HR, 0.76; 95% CI, 0.63-0.92; *P* = .02) (Table 2). When considering the relative associations of cataract extraction, additional education, White race, smoking history, sex, and *APOE* genotype with dementia risks, the only covariate that was more protective than cataract surgery was not having an *APOE* e4 allele (Figure 2).

**Table 2.** Association of Eye Surgery as a Time-Varying Exposure and Subsequent All-Cause Dementia as the Outcome Among People With Cataract Diagnosis

Model^a^	Model description	Hazard ratio (95% CI)		
		Surgery exposure (time varying)	Time since surgery^b^	
			>0 to 5 y	>5 y
Model 1^c^	Primary model	0.71 (0.62-0.83)	0.68 (0.56-0.81)	0.76 (0.63-0.92)
Sensitivity analyses^d^				
1a	Omit 1994-1996 enrollment cohort	0.52 (0.39-0.69)	0.47 (0.34-0.66)	0.63 (0.59-0.95)
1b	Exclude surgery 2 y prior to censoring	0.57 (0.48-0.66)	0.44 (0.35-0.55)	0.70 (0.58-0.85)
1c	Adjust for additional covariates	0.75 (0.65-0.88)	0.72 (0.59-0.86)	0.80 (0.66-0.97)
1d	Consider only incident cataract cases	0.70 (0.56-0.87)	0.69 (0.53-0.89)	0.72 (0.54-0.95)
1e	Incident cataract cases, controlling for CASI at time of cataract diagnosis	0.70 (0.57-0.87)	0.69 (0.53-0.89)	0.72 (0.55-0.96)
1f	Adjust recent vs long-term threshold to 2-y window	NA	0.60 (0.46-0.79)	0.75 (0.64-0.88)
1g	Adjust recent vs long-term threshold to 10-y window	NA	0.71 (0.61-0.83)	0.72 (0.54-0.97)
Model 2^e^	Marginal structural model with weights for surgery, death, and dropout to account for healthy patient bias	0.71 (0.60-0.85)	0.73 (0.61-0.88)	0.66 (0.51-0.86)
Sensitivity analyses				
2a	Marginal structural model with weights for surgery only	0.73 (0.62-0.87)	0.75 (0.62-0.90)	0.70 (0.54-0.90)
2b	Adjust for additional covariates	0.72 (0.61-0.86)	0.73 (0.60-0.88)	0.70 (0.54-0.91)
Model 3^f^	Glaucoma surgery	1.08 (0.75-1.56)	1.15 (0.72-1.83)	1.00 (0.59-1.70)

Abbreviations: ACT, Adult Changes in Thought; BMI, body mass index (calculated as weight in kilograms divided by height in meters squared); CASI, Cognitive Abilities Screening Instrument; IADL, instrumental activity of daily living; NA, not applicable.

^a^ All models use age as the time axis and adjust for years of education, self-reported White race, and past or current smoking and are stratified by any apolipoprotein E ε4 alleles, sex (to meet proportional hazards assumptions), and age at first cataract diagnosis (<68, 68-71, 72-76, and ≥77 years).

^b^ A time-dependent covariate that is set at the first category (0 to 5 years) for the first 5 years after surgery and then to the second category (>5 years) after that.

^c^ During follow-up of 23 554 person-years, there were 853 cases of incident dementia; 504 cases occurred during the 15 941 person-years before or without cataract surgery (0.033 per person-year), and 320 occurred during the 7603 person-years after surgery (0.042 per person-year).

^d^ Sensitivity analyses include results after excluding the original ACT cohort recruited between 1994 and 1996 (model 1a; n = 2868); ignoring any cataract surgical procedure occurring within 2 years of dementia diagnosis or censoring (model 1b); adding these additional covariates to model 1: diabetes, systolic blood pressure, hypertension, heart disease, cardiovascular disease, BMI, self-rated health, Charlson Comorbidity Index, number of activities of daily living and IADL limitations, at least 15 minutes of physical activities 3 times a week, performance-based physical function scores, Centers for Epidemiologic Studies Depression Scale scores, retirement status, and difficulty with near and distance vision (model 1c); excluding data from people with prevalent cataract diagnosis at time of ACT study entry (model 1 day; n = 1556); including baseline CASI score at the time of diagnosis (model 1e; n = 1556); limiting the recent cataract category to a 2-year window (model 1f); and limiting the recent cataract category to a 10-year window (model 1g).

^e^ Model 2 used stabilized time-varying weights to adjust for the probability of surgery, death, and dropout (eMethods 4 and eTables 2, 3, and 4 in the Supplement). Model 2a used stabilized time-varying weights in a marginal structural model adjusting only for the probability of surgery (eMethods 4 and eTables 2-4 in the Supplement). Model 2b is model 2 additionally controlled for diabetes, systolic blood pressure, hypertension, heart disease, cardiovascular disease, BMI, self-rated health, Charlson Comorbidity Index, number of activities of daily living and IADL limitations, at least 15 minutes of physical activities 3 times a week, performance-based physical function scores, Center for Epidemiologic Studies Depression Scale scores, retirement status, and self-reported difficulty with near and distance vision.

^f^ Model 3 is survival analysis with the same covariates and dementia outcome as in model 1 but with the exposure of interest as history of glaucoma surgery instead of cataract surgery (n = 728) and risk starting with first glaucoma diagnosis. During 5029 person-years of follow-up, there were 230 cases of incident dementia; 194 cases occurred during the 4497 person-years before or without glaucoma surgery (0.043 per person-year), and 36 cases occurred during the 553 person-years after surgery (0.062 per person-year).

### Secondary Analyses

Models excluding the 1994-1996 cohort found a lower adjusted HR for dementia (0.52; 95% CI, 0.39-0.69; *P* < .001 (Table 2, model 1a). Models counting the first 2 years after cataract surgery as unexposed person-time for the development of dementia found a lower adjusted HR for dementia (0.57; 95% CI, 0.48-0.66; *P* < .001) (Table 2, model 1b). Results were similar in models including the list of confounding factors delineated in the Methods section (Table 2, model 1c) and in marginal structural models incorporating weights to account for healthy patient bias (Table 2, models 2, 2a, and 2b).

Restricting analyses to participants with incident cataracts showed similarly lower risks (Table 2, model 1d). Controlling for CASI score at the time of cataract diagnosis or altering the recent vs established surgery thresholds did not substantially alter HR estimates (Table 2, models 1e, 1f, and 1g). Adjusting for health care utilization rates did not change the primary model (HR for any surgery was 0.70; 95% CI, 0.60-0.82; *P* < .001).

### AD Dementia Risks and Analyses of Glaucoma Surgery

Results for AD dementia were similar to those for all-cause dementia (eResults 3 and eTables 6 and 7 in the Supplement). In models of 728 participants who received a diagnosis of glaucoma (eTable 8 and eResults 4 in the Supplement), glaucoma surgery (105 participants [14%]) was not associated with a decreased risk of dementia (HR, 1.08; 95% CI, 0.75-1.56; *P* = .68; Table 2, model 3) during the follow-up of 5029 person-years.

## Discussion

Based on 23 554 person-years of follow-up data for 3038 study participants with cataracts, the risk of developing all-cause dementia in participants who underwent cataract extraction was significantly lower than that for people who did not undergo cataract surgery (HR, 0.71; 95% CI, 0.62-0.83; *P* < .001). This difference was still significant after controlling for multiple confounders and when using marginal structural models to account for healthy patient bias. In contrast to cataract extraction, we did not find lower risk associated with glaucoma surgery among people with glaucoma.

Several studies have shown associations between sensory impairment and cognitive decline.^29,30,31^ Sensory impairment may contribute to social isolation and decreased cognitive stimulation, which may increase the risk of dementia.^1,32^ However, prior interventional studies on the association of reversing visual or hearing impairments with reducing dementia risk have shown mixed results.^1,3,33,34^ One of the most challenging issues specific to the procedure-based epidemiological question is an immortal time bias. We have specifically addressed this challenge in our study by treating surgery as a time-varying exposure.^16,17,18,19,20^

Associations between cataract extraction and dementia development have been similarly conflicting. Most studies did not use research-quality dementia identification,^23,25^ which may have contributed to varying results. In a cross-sectional study of 2764 Japanese participants (mean [SD] age, 76.3 [4.8] years; 52.6% male), the cataract surgery group (n = 668 [24.2%]) had a lower odds ratio for mild cognitive impairment than the group without cataract surgery (n = 2096 [75.8%]) (odds ratio, 0.78; 95% CI, 0.64-0.96; *P* = .02), but no difference was found for dementia (odds ratio, 1.10; 95% CI, 0.75-1.62; *P* = .64).^11^ A retrospective study with 10 years of follow-up using the Taiwan National Health Insurance Research Database of 113 123 patients with cataract showed a lower HR for dementia among people with cataract surgery (HR, 0.74; 95% CI, 0.75-0.79; *P* < .001).^12^ Although similar to our results, that study relied on dementia diagnoses from usual care and lacked data on factors such as *APOE* genotype, years of education, and smoking.

Cataract extraction could appear to have a protective association owing to healthy patient bias, in which participants who underwent cataract surgery were healthier and at lower risk of dementia. We performed several analyses to address this potential bias. Furthermore, we evaluated glaucoma surgery, which, unlike cataract surgery, does not improve vision. Our findings in all of these analyses were consistent with a cataract extraction–specific association with dementia risk, potentially because of improvements in vision and visual function. We also considered the possibility of more health-conscious participants having a higher level of health care utilization and thus being more inclined to undergo cataract surgery affecting dementia development. However, adjustment for health care utilization had no effect on our models. It is possible that we had insufficient power in the glaucoma analysis, but this scenario is unlikely given that the point estimate of glaucoma surgery was very close to null (HR, 1.08; 95% CI, 0.75-1.56).

Low vision from cataract may impair performance on vision-dependent screening tests for dementia, and scores may improve after cataract surgery owing to better vision.^35^ In our study, anyone with visual impairment noted by a trained study staff member underwent the full standardized dementia evaluation, which included extensive vision-independent cognitive assessment methods. Theoretically, individuals with mild dementia could possibly be missed if they scored barely above our screening threshold and their vision was good enough that they were not automatically referred on the basis of low vision, whereas a similar person with the same mild dementia but with very poor vision would be automatically referred and have their dementia identified by a study staff member. However, very few people had dementia detected on the basis of referral due to vision concerns; thus, it is unlikely that this theoretical concern is driving the results we observed. In addition, our results did not change when we controlled for participants’ self-reported difficulty with distance or near vision at the time of cataract diagnosis, which provides further reassurance that vision impairment was not driving our dementia findings.

Several hypothesized mechanisms may underlie the association between cataract extraction and dementia risk. Visual impairment may lead to psychosocial difficulties, withdrawal from social interactions, and reduction in activity or exercise, all of which are associated with cognitive decline.^1,36^ Cataract-related visual impairment may decrease neuronal input, potentially accelerating neurodegeneration or magnifying the effect of neurodegeneration through cortical atrophy. The visual cortex undergoes structural changes with vision loss.^37,38^ For patients with neovascular age-related macular degeneration, vision loss was associated with visual cortex atrophy during a 5-year follow-up,^39^ and an increase in gray matter volume has been observed after cataract surgery.^40^ Finally, compensation for visual input deficit may increase cognitive load and exacerbate cognitive decline.^41^

Lower risk for developing dementia following cataract extraction may also be associated with increased quantity and quality of light. Intrinsically photosensitive retinal ganglion cells (ipRGCs), which are exquisitely sensitive to short-wavelength (blue) light, have been shown to be associated with cognitive function, circadian rhythm, and AD.^42^ The ipRGCs project to multiple areas of the brain, and their excitation may trigger widespread cortical activity.^43^ The yellow hue of age-related cataracts blocks blue light. Thus, another potential mechanism for which cataract extraction is associated with decreased risk of dementia is the facilitation of ipRGC stimulation by blue light.

We must acknowledge that our results could be explained by unmeasured or residual confounding, like any observational study. There were some suggested differences between people who underwent cataract surgery and people who did not, but controlling for a broad spectrum of factors underlying these differences between people with and people without surgery did not meaningfully change our findings. We also compared findings for cataract surgery to those for glaucoma surgery in the same cohort. In essence, we used glaucoma surgery as a negative control. Admittedly, the 2 surgical procedures have different indications, so the comparison is only an approximate approach to address the possibility of healthy patient bias. Nonetheless, the present study may be the highest-quality evidence we will have to address the underlying question because there could be ethical and practical concerns regarding a trial that delays cataract surgery.

### Strengths and Limitations

Our study has several strengths. First, it was based on a prospective, community-based observational cohort of more than 3000 participants with 23 554 person-years of follow-up recruited when individuals were dementia free and systematically followed up until dementia development. Second, more than 98% of the ACT study cohort visited eye care clinicians at least once, with a median of 21 encounters (IQR, 10-37).^44^ Our study has the advantage of a resource-rich, integrated health care delivery system setting in which everyone had access to comprehensive eye care. We can thus disentangle the effects of cataract extraction from access to care that could include cataract surgery. Third, dementia diagnoses were made by a panel of experts using research criteria. Given the strong implications in the care of older adults that were derived from our findings, the reliable dementia diagnoses used in our study are crucial. Fourth, we thoroughly investigated the possibility of healthy patient bias and potential confounders. Adding baseline CASI score at the time of cataract diagnosis did not change our results.

Several limitations exist. Cataract diagnosis and surgery were based on diagnosis and procedure codes available from electronic medical records, and we did not have ophthalmic clinical data, such as visual acuity or cataract severity. Coding errors cannot be ruled out, although such errors should bias toward the null. We evaluated only the participant’s first cataract surgery and do not know whether any surgery in the contralateral eye impacted dementia risk. Reverse causation is a potential concern. People with early cognitive problems may be less conscious of vision issues and thus may undergo cataract surgery at a later age. Although this possibility cannot be completely ruled out, when we excluded cataract operations in the 2 years prior to dementia diagnoses, we found that the protection associated with surgery was even stronger. Furthermore, because we had access to electronic health record data starting only in 1993, 1 year prior to the original cohort recruitment, there may have been an underestimation in the cataract diagnosis duration in some participants. Thus, we repeated our analysis, excluding the original cohort, and found similar rates. The lack of biomarker-based AD may be considered a limitation. However, our study is longitudinal in nature and primarily interested in clinical dementia. Finally, our study population was composed primarily of persons of self-reported White race and thus may not be representative of other populations.

### Conclusions

The results of our cohort study showed that cataract extraction had a significant association with lower risk of developing dementia among adults 65 years of age or older. These results have implications for the care of older persons who are uniquely at higher risk for both impaired vision due to cataract and impaired cognition due to neurodegeneration observed in age-related dementia. Given the substantial degree by which cataract extraction is associated with lower risk of dementia and its persistent effect beyond 10 years, the improvement in quality of life for the affected individuals and their family is likely considerable. Further studies on the mechanisms by which cataract extraction may affect dementia risk are warranted.

## Notes

Supplement. **eMethods 1.** Inclusion and Exclusion Criteria **eMethods 2.** Neuropsychological Battery and Dementia Detection Protocol **eMethods 3.** Variables **eMethods 4.** Marginal Structural Models **eResults 1.** Demographic and Health Variables **eResults 2.** Demographic and Health Variables, Incident Cataract **eResults 3.** Study Population, Alzheimer Disease Dementia Risks **eResults 4.** Demographic and Health Variables in Participants With Glaucoma Diagnosis **eTable 1.** *International Classification of Diseases, Ninth Revision, Clinical Modification* (*ICD-9-CM*); *International Classification of Diseases, Tenth Revision, Clinical Modification* (*ICD-10-CM*); and *Current Procedural Terminology* (*CPT*) Codes Used in the Analysis **eTable 2.** Area Under the Receiver Operating Characteristics (ROC) Curves for Obtaining Weights for the Marginal Structural Models **eTable 3.** Description of the Stabilized Weights for the Marginal Structural Models **eTable 4.** Marginal Structural Models (MSM) for All-Cause Dementia With No Weights and With Un-Winsorized Weights **eTable 5.** Demographic and Health Variables at First Cataract Diagnosis by Later Surgery Status, Among Those Diagnosed With Cataracts After Study Enrollment **eTable 6.** Survival Analysis and Marginal Structural Model for Developing Alzheimer Disease Dementia, Comparing the Cataract Surgery and No Surgery Groups **eTable 7.** Marginal Structural Models (MSM) for Alzheimer Disease (AD) Dementia With No Weights and With Un-Winsorized Weights **eTable 8.** Demographic and Health Variables at First Glaucoma Diagnosis by Later Surgery Status, Among Those Diagnosed With Glaucoma
eReferences.
