# Supplementary material for: Association Between Cataract Extraction and Development of Dementia
Source: JAMA Intern Med. 2021 Dec 6;182(2):134–41. doi: 10.1001/jamainternmed.2021.6990 (PMC8649913; doi:10.1001/jamainternmed.2021.6990)
Supplement: Supplement. — eMethods 1. Inclusion and Exclusion Criteria eMethods 2. Neuropsychological Battery and Dementia Detection Protocol eMethods 3. Variables eMethods 4. Marginal Structural Models eResults 1. Demographic and Health Variables eResults 2. Demographic and Health Variables, Incident Cataract eResults 3. Study Population, Alzheimer Disease Dementia Risks eResults 4. Demographic and Health Variables in Participants With Glaucoma Diagnosis eTable 1. International Classification of Diseases, Ninth Revision, Clinical Modification (ICD-9-CM); International Classification of Diseases, Tenth Revision, Clinical Modification (ICD-10-CM); and Current Procedural Terminology (CPT) Codes Used in the Analysis eTable 2. Area Under the Receiver Operating Characteristics (ROC) Curves for Obtaining Weights for the Marginal Structural Models eTable 3. Description of the Stabilized Weights for the Marginal Structural Models eTable 4. Marginal Structural Models (MSM) for All-Cause Dementia With No Weights and With Un-Winsorized Weights eTable 5. Demographic and Health Variables at First Cataract Diagnosis by Later Surgery Status, Among Those Diagnosed With Cataracts After Study Enrollment eTable 6. Survival Analysis and Marginal Structural Model for Developing Alzheimer Disease Dementia, Comparing the Cataract Surgery and No Surgery Groups eTable 7. Marginal Structural Models (MSM) for Alzheimer Disease (AD) Dementia With No Weights and With Un-Winsorized Weights eTable 8. Demographic and Health Variables at First Glaucoma Diagnosis by Later Surgery Status, Among Those Diagnosed With Glaucoma eReferences. [file jamainternmed-e216990-s001.pdf]

Supplementary Online Content

Lee CS, Gibbons LE, Lee AY, et al. Association between cataract extraction and development of dementia. *JAMA Intern Med*. Published online December 6, 2021.
doi:10.1001/jamainternmed.2021.6990

eMethods 1. Inclusion and Exclusion Criteria
eMethods 2. Neuropsychological Battery and Dementia Detection Protocol
eMethods 3. Variables
eMethods 4. Marginal Structural Models
eResults 1. Demographic and Health Variables
eResults 2. Demographic and Health Variables, Incident Cataract
eResults 3. Study Population, Alzheimer Disease Dementia Risks
eResults 4. Demographic and Health Variables in Participants With Glaucoma Diagnosis
eTable 1. *International Classification of Diseases, Ninth Revision, Clinical Modification* (*ICD-9-CM*); *International Classification of Diseases, Tenth Revision, Clinical Modification* (*ICD-10-CM*); and *Current Procedural Terminology* (*CPT*) Codes Used in the Analysis
eTable 2. Area Under the Receiver Operating Characteristics (ROC) Curves for Obtaining Weights for the Marginal Structural Models
eTable 3. Description of the Stabilized Weights for the Marginal Structural Models
eTable 4. Marginal Structural Models (MSM) for All-Cause Dementia With No Weights and With Un-Winsorized Weights
eTable 5. Demographic and Health Variables at First Cataract Diagnosis by Later Surgery Status, Among Those Diagnosed With Cataracts After Study Enrollment
eTable 6. Survival Analysis and Marginal Structural Model for Developing Alzheimer Disease Dementia, Comparing the Cataract Surgery and No Surgery Groups
eTable 7. Marginal Structural Models (MSM) for Alzheimer Disease (AD) Dementia With No Weights and With Un-Winsorized Weights
eTable 8. Demographic and Health Variables at First Glaucoma Diagnosis by Later Surgery Status, Among Those Diagnosed With Glaucoma
eReferences.

This supplementary material has been provided by the authors to give readers additional information about their work.

© 2021 Lee CS et al. *JAMA Internal Medicine*.

eMethods 1. Inclusion and Exclusion Criteria

Detailed study methods have been published previously.[1,2] Briefly, the ACT study began in 1994-96 and is an ongoing, population-based, prospective cohort study of older adults recruited from Kaiser Permanente Washington (KPW) membership rolls and followed until dementia development. KPW is a Washington state comprehensive health care delivery system established in 1947 in the United States. The ACT study includes over 5,500 adults and 1,270 incident cases of Diagnostic and Statistical Manual of Mental Disorders, 4th Edition (DSM-IV) dementia.[1] Participants are ≥65 years old and dementia free at enrollment. At enrollment and during biennial visits, participants receive standardized cognitive screening tests, brief physical evaluations, and medical history and risk factor assessments.[2,3] We included all ACT participants who had a diagnosis of cataract before onset of dementia and at least one study visit after cataract diagnosis. Participants with concurrent other ophthalmic diseases were not excluded. We also included ACT participants with diagnosis of glaucoma, and their surgery records, for secondary analyses. Patients who had cataract surgery before ACT enrollment or without *APOE* genotype data were excluded. Self-reported race information is collected for demographic analysis. All participants gave informed written consent. Participants were followed from cataract diagnosis (incident or prevalent) until the 2018 data freeze.

© 2021 Lee CS et al. *JAMA Internal Medicine*.

eMethods 2. Neuropsychological Battery and Dementia Detection Protocol

Participants are evaluated biennially with the Cognitive Abilities Screening Instrument (CASI), which ranges from 0-100, with higher scores better.[4] Participants with CASI scores ≤ 85 undergo a standardized diagnostic evaluation, including physical and neurologic examinations, extensive medical records review, and a neuropsychological test battery.[5]

The following tests are included in the dementia psychometric battery: clock drawing,[6] verbal fluency,[7] the Mattis Dementia Rating Scale,[8] the Boston naming test,[7] verbal paired associations and recall, logical memory and recall,[9] Word List Memory,[7] Constructional Praxis and recall,[7] Trails A and B,[10] and Information and Comprehension subtest items.[9] At a consensus conference, all clinical data are reviewed. Clinical laboratory and imaging studies of subjects diagnosed with dementia were reviewed to assign dementia subtype (e.g. Alzheimer disease dementia, vascular dementia, normal pressure hydrocephalus, etc.). If these results are unavailable from medical records, they are requested to be ordered by the delivery system, results are obtained, and then reviewed again at a subsequent consensus conference.

Since 1986, dementia onset in the Adult Changes in Thought (ACT) study was assigned to a date halfway between the prior evaluation and the exam that diagnosed dementia. The incidence rates of dementia within this study are consistent with those found worldwide,[1] supporting the validity of our case definitions. Forest plots of associations between alleles of single nucleotide polymorphisms and dementia from Alzheimer disease suggest similar strength of association for cases and controls ascertained by the ACT study as those from more than a dozen other research studies of dementia from Alzheimer disease.[11]

The ACT neuropsychological battery evaluates multiple cognitive domains. Assessment of executive functioning (Mattis Initiation and Conceptualization scales, comprehension, Trails, fluency, and clock drawing) would aid in identification of vascular cognitive impairment / vascular dementia and frontotemporal dementia. Assessment of spatial ability (clock and Mattis construction) helps aid the diagnosis of Lewy body dementia and Parkinson's disease with dementia. The diagnostic process is based on both psychometric test results and historical and clinical elements. An expert consensus of clinicians and neuropsychologists use all available data to determine the diagnosis of dementia, using standardized definitions from the Diagnostic and Statistical Manual, 4th Edition (DSM-IV) for dementia[12] and McKhann et al. criteria for probable or possible Alzheimer disease dementia.[13]

For any participants who may have issues with vision, large print visuals are used for administering the CASI. Additional, optional cognitive evaluations such as Blessed[14] and Jorms[15] are used to evaluate whether CASI scores may be impacted by sensory impairment. Thus, on rare occasions when the participants cannot see the large print visuals for CASI and/or are suspected to have significant visual impairment during the cognitive evaluation or per family member, they are referred for a full dementia evaluation since their cognitive functioning cannot be validly assessed by the CASI alone. To date this has occurred rarely in ACT. There have been a total of 17 referrals due to significantly impaired vision, which resulted in 10 dementia diagnoses. Less than 1% of all dementia referrals and <1% of all dementia cases were identified due to significantly impaired vision as opposed to low scores on the CASI.

© 2021 Lee CS et al. *JAMA Internal Medicine*.

eMethods 3. Variables

The following variables were based on self-reported medical history at enrollment and were updated at each biennial follow-up: smoking, hypertension, congestive heart failure, diabetes, history of cardiovascular disease (myocardial infarction, angina, coronary artery bypass grafting [CABG], or angioplasty), and cerebrovascular disease (stroke, transient ischemic attack, or carotid endarterectomy). Participants were asked whether they had any difficulty with distance or near vision at each follow-up even with corrective lenses. Health utilization rate was assessed as the number of ambulatory visits per year in the 5 years prior to cataract diagnosis. For those with < 5 years of EHR before cataract diagnosis, the rate was calculated based on the number of days enrolled at KPW. Ophthalmic diagnoses and procedures were extracted from participants' electronic medical records (EMR), available from 1993 onwards (1 year prior to ACT's initial enrollment period).(**eTable 1**) In secondary analyses, we excluded the 1994-1996 enrollment cohort to avoid potential misclassification from diagnoses or surgeries preceding EMR data.

We also extracted glaucoma diagnosis and surgery records. Glaucoma and cataract surgeries have similar eligibility considerations in terms of comorbidities, but glaucoma surgery does not restore vision. Cataract and glaucoma diagnoses and surgery records were based on International Classification of Diseases (ICD)-9-Clinical Modification (CM), ICD-10-CM, and Current Procedural Terminology (CPT) codes. (eTable 1)

© 2021 Lee CS et al. *JAMA Internal Medicine*.

eMethods 4. Marginal Structural Models

It is possible that the observed apparent protective association we found between surgery and dementia risk is an artifact as those who get surgery may be on average generally healthier and have fewer risk factors for dementia compared to people who do not get surgery. This possibility can be referred to as “healthy patient bias”.

There are several analytic approaches to address this concern. The first is incorporating terms for potential confounders in the primary models. The second is to specifically model how likely people were to have surgery and incorporate those results in models of the effects of surgery on dementia risk. And a third possibility is to examine data from some other procedure that should have similar healthy patient bias effects but that is not hypothesized to have an impact on the mechanism(s) postulated by which cataract surgery in particular is (are) thought to impact dementia and Alzheimer disease dementia risk.

We used all of these strategies. In this Supplemental Text we provide more information on the weighting approach incorporated in the second strategy.

Ideally we would have had a sample in which surgery itself was unrelated to dementia risk, so any relationship between cataract surgery and dementia risk would be due to the improvements in vision and/or other visual function due to the cataract surgery, and not at all because of factors that differed between people who received cataract surgery and those who did not (i.e. healthy patient bias). For this to be true, the decision on whom to perform cataract surgery must be unrelated to factors that confound the relationship between cataract surgery and dementia risk.

If factors that are associated with selection for cataract surgery that confound the relationship between cataract surgery and dementia risk due to factors associated with selection for cataract surgery could be entirely captured at cataract diagnosis, we would use those variables as covariates in the model, and the adjusted hazard for the association between cataract surgery and dementia risk would not reflect healthy patient bias. Unfortunately, factors one could readily imagine would be associated with both selection for cataract surgery and dementia risk are time-varying, such as factors reflecting overall health or fragility. And it is established that adding time-varying covariates to a main model can produce incorrect causal inference due to treatment-confounder feedback.[16] In this setting, we need a strategy that can incorporate time-varying factors that could correctly account for selection for cataract surgery.

For this reason, we used marginal structural models (MSM)[17,18] which are able to account for time-varying confounders of the relationship between cataract surgery and dementia risk. The MSM approach uses weights to create a pseudo population in which there is no healthy patient bias, and performs analyses in that pseudo population of the (unconfounded) relationship between cataract surgery and dementia risk. Intuitively, the weights for the pseudo population emphasize (upweight) people who were less likely to receive surgery but did anyway, and also people who were highly likely to receive surgery but did not.

We start with unstabilized weights. For those receiving surgery, their weight is the inverse of the probability of surgery, and for those not receiving surgery, it is the inverse of (1 - the probability of surgery). Applying unequal weights to the outcome model increases the variance of the estimates in the outcome model. The impact of the weights on the variance is proportional to the variance of the weights (i.e., if everyone's weight is close to 1, the impact is minimal, but if some people have weights much higher than 1 while others have weights much lower, the variance of estimates in the outcome model when weighted will be substantially inflated). Stabilizing the weights reduces the variance of the weights, and therefore reduces the impact of weighting on the variance of the outcome model.[17–19]

Stabilized weights include a numerator and a denominator. The denominator incorporates probability of selection for surgery using both time invariant and time varying factors, while the numerator incorporates probability for selection for surgery using only time invariant factors. Time invariant factors are also included in the main outcome model; otherwise, they would not be controlled for as they are in both the numerator and denominator. Time varying factors are not included in the main outcome model due to treatment-confounder feedback mentioned earlier.[16]

For both the numerator and the denominator for the stabilized weights, we chose variables that were plausible predictors of both cataract surgery and dementia risk. In the numerator, we predicted the estimated probability of surgery using the following time-constant variables: age of cataract diagnosis, education, self-reported White race, smoking history at time of diagnosis, presence of ≥1 *APOE* ε4 allele, and sex. The denominator included all of those terms and additionally included the following time-varying covariates, all of which were updated at the time of the participant's most recent ACT study visit: Cognitive Abilities Screening Instrument (CASI) scores, several self-reported conditions including diabetes, hypertension, heart disease, and cardiovascular disease, measured systolic blood pressure and body mass index, self-rated health, Charlson comorbidity index (CCI), number of self-reported activities of daily living (ADLs and Instrumental ADLs) limitations, self-reported performance of at least 15 minutes of physical activity three times a week, performance-based physical function scores, Center for Epidemiologic Studies Depression Scale (CESD) scores, and self-reported retirement status. These logistic models were estimated defining each month of follow-up as a time interval and estimating odds of cataract surgery in that interval, censoring individuals after first surgery. All covariates met positivity assumptions.

We used the same methods and variables to construct stabilized weights for death, and for attrition, and the final weights in our primary models (**Table 2** main text, Models 2 and 2b) were the product of the surgery, death and attrition weights. The area under the receiver operating characteristic (ROC) curves for the numerators and denominators of each of these weights are in **eTable 2**. In predicting treatment (probability of surgery), the area under the ROC curve for the numerator model, which uses only time-constant variables, was 0.58. The area under the ROC curve for the denominator model which adds time-varying variables was 0.62. The similarity between those areas under the ROC curves suggests that time-varying confounding is unlikely to be large. The factors driving surgery are largely unrelated to the many measures we had for health, cognitive and physical function, and mood. This indicates that our simpler, unweighted primary model (**Table 2**, Model 1) may be legitimate.

Note that practice is to Winsorize the weights,[17] that is to recode the values in the top 1% to the 99th percentile (here 2.29) and the values in the bottom 1% to the first percentile (here 0.19). The few observations resulting in very small combined stabilized weights, under 0.2, were for the visits of 12 people at least 9 years after diagnosis (mean 16), when they were 88-101 years old. The combined stabilized weights over 5 were also from later visits, 32 participants with mean age 88 at the time of the visit. The stabilized weights summarized in **eTable 3** were used in the MSM in **Table 2** and in **eTables 4**, **6,** and **7**.

Time-dependent weights require discrete-time survival models.[18] We modeled the probability of dementia each month, weighted based on the probability the individual received the treatment they actually received in the prior month, using stabilized weights. Outcome models were estimated using a logit link in a weighted generalized linear regression model with sandwich variance estimators, in each month of observation and censoring at dementia, death, or last visit. This model included a cubic spline to account for time since enrollment. Because a dementia diagnosis in any month is rare, the logit model approximates a hazard.

The results from MSM models using no weights, as well as weights that were not Winsorized, with some models from **Table 2** (main text) included for comparison are shown in **eTable 4**. For all-cause dementia, results from the unweighted MSM were very close to those from Model 1, our primary outcome, for any surgery versus none, though the effects for time since surgery were closer to each other than in Model 1. Results from models using stabilized weights based on the probability of surgery were very similar (Model 2a). Results from models using the combined

© 2021 Lee CS et al. *JAMA Internal Medicine*.

stabilized weights for surgery, death and attrition without Winsorizing showed markedly stronger protective effects for surgery, which may reflect the extreme values for some of the weights as reported in **eTable 3**. Results from the combined stabilized and Winsorized weights for surgery death, and attrition were consistent with those from the other models.

eResults 1. Demographic and Health Variables

Our primary analyses include people diagnosed with cataracts before and after ACT study enrollment. The demographic and health information at first cataract diagnosis, or at baseline if first cataract diagnosis was before baseline, by later surgery status, was presented in **Table 1** (main text). Higher proportions of those with surgery were female, younger at ACT study entry and at first cataract diagnosis, and had self-rated good health, and lower systolic blood pressure, compared to those who did not have cataract surgery. A total of 2752 participants were White (91%), 99 were Asian (3%), 91 were Black/African American (3%), and 96 were mixed race, American Indian or Alaskan native, other, or unknown (3%). Forty-nine percent of cataract diagnoses preceded ACT study enrollment. (**eTable 5**) A small difference in the rate of health utilization in the five years preceding cataract diagnosis was found between the two groups; the median number of ambulatory visits per year was 6.5 (Interquartile range [IQR] 3.6 – 11.0) for those who had cataract extraction and 6.2 (IQR 3.0 – 10.8) for those who did not (Wilcoxon rank-sum p=0.02). Censoring of participants occurred as follows: 893 participants died, 362 dropped out (due to no visit within 2.5 years of previous visit), and 1783 were censored at their last study visit (either because of dementia or data freeze).

eResults 2. Demographic and Health Variables, Incident Cataract

Here we present the same information but limited to those diagnosed with cataracts after ACT study enrollment. (Model 1d in **Table 2** and **eTable 6**) A total of 1,556 participants developed cataract after the ACT study enrollment, referred to as incident cataract. The demographic and health variables of the participants who developed incident cataract are shown in **eTable 5**. Slightly less than half (46%) of the participants with incident cataract had surgery. As in the full cohort, people with surgery were more likely to be female and with lower systolic blood pressure. They were also less likely to rate their health poor or fair (11% vs 14%), though the difference was not statistically significant in this smaller sample. But in this group, those with surgery had similar age at first cataract diagnosis and years of education.

© 2021 Lee CS et al. *JAMA Internal Medicine*.

eResults 3. Study Population, Alzheimer Disease Dementia Risks

Of the 5,546 ACT participants, 4,508 had *APOE* genotype data. Of these, 3,421 had been diagnosed with a cataract before AD dementia onset and 3038 did not have surgery before ACT study baseline. All 3,038 had at least one study visit after cataract diagnosis (total person-year 23,554; average 7.8 years/person) and were thus included in the study. There were 709 incident AD dementia cases. Participants who underwent cataract surgery were significantly less likely (hazard ratio [HR] 0.72, 95% confidence interval [CI] 0.61, 0.85, p<0.001) to develop AD dementia than comparable patients who did not undergo cataract surgery after adjusting for education, self-reported White race, smoking history, and stratifying by *APOE* genotype, sex, and age at first cataract diagnosis. This effect was more prominent during the first 5 years following cataract surgery (HR 0.65, CI 0.53, 0.80, p<0.001) than after the 5-year period (HR 0.81, CI 0.66, 0.99, p=0.039). (**eTable 6; Figure 2B**)

Secondary Analyses with Alzheimer disease dementia

Omitting data from the original ACT cohort enrolled 1994-1996 in the analyses resulted in even lower HR (0.50, 95%CI 0.37, 0.70, p<0.001) of developing AD dementia in the cataract surgery group compared to the no surgery group.(**eTable 6**, Model 1a) To limit potential bias due to the possibility that participants with early signs of not yet diagnosed dementia being less likely to have cataract surgery, we ignored any surgery in the 2 years before the diagnosis of AD dementia. Again, the protective effect of surgery on AD dementia risk appeared to be even stronger in these models, with an overall HR of 0.60 (95%CI 0.51, 0.70, p<0.001), with a HR of 0.43 (95%CI 0.34, 0.55, p<0.001) for recent surgery (defined as less than 5 years from cataract diagnosis) and a HR of 0.73 (95%CI 0.61, 0.88, p=0.001) for those who had cataract surgery greater than 5 years after cataract diagnosis. (**eTable 6**, Model 1b) Potential healthy patient bias was controlled by adjusting for an extensive list of factors mentioned in the Methods (**eTable 6**, Model 1c) and also by creating marginal structural models where participants were weighted to account for their health status. (**eTable 6**, Model 2) In every case, results were similar to those from our primary model 1. Restricting our analysis to participants with incident diagnosis of cataract following ACT study enrollment showed similar results. (**eTable 6**, Model 1d) Modifying our cut-off to define recent vs established surgery groups did not have a substantial impact on the HR: the overall HRs of developing AD dementia were lower when we evaluated the short term effect vs the long term effect defined as less or more than 2 years, or less or more than 10 years. (**eTable 6**, Models 1e and 1f) As we had hypothesized, glaucoma surgery did not have any association with AD dementia risk (HR 1.25, 95%CI 0.85, 1.86, p=0.26) regardless of time since surgery. (**eTable 6**, Model 3)

eTable 7 includes the results from marginal structural models using no weights, as well as weights that were not Winsorized, with some models from **eTable 6** included for comparison. The unweighted MSM was very close to Model 1, our primary outcome, for any surgery versus none, though the effects for time since surgery were closer to each other than in Model 1. Applying stabilized weights based on the probability of surgery did not produce much change (Model 2a). Using the combined stabilized weights for surgery, death and attrition without Winsorizing resulted in markedly stronger protective effects for surgery, but note in **eTable 3** that there were some extreme values for the weights. Winsorizing weights for the top and bottom 1% produced results consistent with the other models.

eResults 4. Demographic and Health Variables in Participants With Glaucoma Diagnosis

Our third approach of addressing healthy patient bias was to examine data from some other procedure that should have similar healthy patient bias effects but that is not hypothesized to have an impact on the mechanism(s) postulated by which cataract surgery in particular is (are) thought to impact dementia and Alzheimer dementia risk. We chose glaucoma surgery as a comparable, outpatient ophthalmic procedure with similar healthy patient bias but glaucoma surgery does not improve vision and/or visual function. As hypothesized, we found no associations between glaucoma surgery and dementia or Alzheimer dementia risks. In **eTable 8**, we report demographic and health information on participants with glaucoma, later by glaucoma surgery group.

A total of 728 participants had a diagnosis of glaucoma and no surgery before ACT study baseline. The demographic and health variables of the participants with glaucoma are shown in **eTable 8**. Two people were missing smoking data so the survival models reported in **Table 2** and **eTable 6** are based on n=726.

A total of 105 participants (14%) underwent glaucoma surgery. There were no significant differences between the glaucoma surgery and no surgery groups except that those who later had surgery were significantly younger at glaucoma diagnosis and more likely to have at least one *APOE* ε4 allele. The proportions of people reporting poor or fair health was 12% in the surgery group and 19% in the no surgery group, a similar difference to that found with cataract surgery (12% vs 15%), but here with a much smaller sample, the difference was not statistically significant. (**eTable 8**)

© 2021 Lee CS et al. *JAMA Internal Medicine*.

eTable 1. ***International Classification of Diseases, Ninth Revision, Clinical Modification*** **(*ICD-9-CM*);** ***International Classification of Diseases, Tenth Revision, Clinical Modification*** **(*ICD-10-CM*); and** ***Current Procedural Terminology*** **(*CPT*) Codes Used in the Analysis**

**Diagnoses**		**ICD-9-CM and ICD-10-CM Codes**
Cataract		366.1*, 366.2*, 366.3*, 366.4*, 366.8*, 366.9*, H25*, H26.1*, H26.2*, H26.3*, H26.8*, H26.9*, H28*
Glaucoma		365.1*, 365.2*, 365.3*, 365.4*, 36.5*, 365.6*, 365.8*, 365.9, H40.1*, H40.2*, H40.3*, H40.4*, H40.5*, H40.6*, H40.8*, H40.9
**Procedures**		**CPT Codes**
Cataract Extraction		66984, 66983, 66982
Glaucoma Surgery		65850, 66150, 66155, 66160, 66170, 66172, 66179, 66180, 66183, 66184, 66185, 66820, 66625, 66710, 66711, 0191T, 0253T, 0376T, 0449T, 0474T

* Asterisks indicate wild cards. For example, 366.1* could be 366.1 exactly but also 366.11, 366.12, 366.13, etc.

© 2021 Lee CS et al. *JAMA Internal Medicine*.

eTable 2. Area Under the Receiver Operating Characteristics (ROC) Curves for Obtaining Weights for the Marginal Structural Models

**Model**	**ROC**
Probability of treatment (surgery)	
Denominator	0.62
Numerator	0.58
Probability of death	
Denominator	0.84
Numerator	0.72
Probability of dropout	
Denominator	0.74
Numerator	0.64

© 2021 Lee CS et al. *JAMA Internal Medicine*.

eTable 3. Description of the Stabilized Weights for the Marginal Structural Models

Stabilized weights	Mean	SD	Min	Max
Exposure[a] only	1.00	0.15	0.30	7.27
Exposure, death and dropout	1.00	1.50	0.04	326.88
Exposure, death and dropout, Winsorized[b]	0.96	0.26	0.19	2.29

[a]Exposure was cataract surgery.
[b]Values in the top 1% were recoded to the 99th percentile; values in the bottom 1% were recoded to the first percentile. SD: standard deviation.

© 2021 Lee CS et al. *JAMA Internal Medicine*.

eTable 4. Marginal Structural Models (MSM) for All-Cause Dementia With No Weights and With Un-Winsorized Weights

Risk ratios and 95% confidence intervals for the association of eye surgery and subsequent all-cause dementia among people with cataract diagnosis. Age was the time axis, starting at baseline if the first cataract diagnosis was before baseline, or at incident cataract diagnosis. These MSM were adjusted for age of cataract diagnosis, sex, education, ≥1 *APOE* ε4 alleles, self-reported White race, and smoking history.

				**Time since surgery**[a]	
**All-Cause Dementia**		Surgery exposure (time-varying)		**> 0 to 5 years**	**Over 5 years**
Model 1 from Table 2:<br>Primary Cox model.		0.71 (0.62, 0.83)		0.68 (0.56, 0.81)	0.76 (0.63, 0.92)
Unweighted MSM:<br>Logit model to approximate a Cox model		0.73 (0.62, 0.85)		0.74 (0.62, 0.87)	0.70 (0.56, 0.89)
Model 2a from Table 2:<br>MSM with surgery weights only		0.73 (0.62, 0.87)		0.75 (0.62, 0.90)	0.70 (0.54, 0.90)
MSM with weights for surgery, death, and attrition, not Winsorized		0.46 (0.27, 0.77)		0.51 (0.31, 0.84)	0.37 (0.21, 0.67)
Model 2 from Table 2:<br>MSM with Winsorized weights for surgery, death, and attrition		0.71 (0.60, 0.85)		0.73 (0.61, 0.88)	0.66 (0.51, 0.86)

[a]A time-dependent covariate which is set at the first category (>0 to 5 years) for the first 5 years following surgery, and then to the second (over 5 years) after that.

© 2021 Lee CS et al. *JAMA Internal Medicine*.

eTable 5. Demographic and Health Variables at First Cataract Diagnosis by Later Surgery Status, Among Those Diagnosed With Cataracts After Study Enrollment

That is, among those with incident cataracts**.** See **Table 1** in main text for demographic and health variables of all participants (incident and prevalent cataract) at first cataract diagnosis.

	**All (N=1,556)**		**Any surgery (N=716)**		**No surgery (N=840)**		
	N or mean	% or SD	N or mean	% or SD	N or mean	% or SD	p-value[a]
Female sex	863	57%	437	61%	446	53%	0.002
Age at ACT study baseline	72.2	5.1	71.9	5.0	72.4	5.2	0.08
Age at first cataract diagnosis	76.1	5.1	76.0	5.0	76.2	5.2	0.26
Education (years)	14.7	3.1	14.7	3.1	14.7	3.1	0.85
Self-reported White race	1,418	91%	656	92%	762	91%	0.53
Any *APOE* ε4 alleles	402	26%	175	24%	227	27%	0.25
Past or current smoker	826	53%	381	53%	445	53%	0.97
Body Mass Index	27.4	4.7	27.2	4.6	27.5	4.8	0.25
Ever reported having diabetes	146	9%	63	9%	83	10%	0.46
Ever reported having hypertension	683	44%	303	42%	380	46%	0.22
Prevalent MI, angina, CABG, or angioplasty	269	17%	113	16%	156	19%	0.15
Ever reported stroke, TIA, or CEA	138	9%	55	8%	83	10%	0.13
CESD score	3.5	4.1	3.5	4.0	3.6	4.1	0.96
Systolic Blood Pressure	138.4	20.5	137.2	20.0	139.4	21.0	0.049
Poor or fair self-rated health	199	13%	78	11%	121	14%	0.07
Any ADL impairments	310	20%	130	18%	180	21%	0.11
Number of ADL impairments	0.3	0.7	0.3	0.6	0.3	0.8	0.08
At least 15 min of activity 3 x per week	1,091	70%	503	70%	588	70%	0.88

[a]Chi-square for dichotomous variables, Wilcoxon rank-sum for continuous variables. SD: standard deviation; ACT: Adult Changes in Thought; MI: myocardial infarction; CABG: coronary artery bypass grafting; TIA: transient ischemic attack; CESD: Center for Epidemiologic Studies Depression Scale; ADL: activity of daily living.

© 2021 Lee CS et al. *JAMA Internal Medicine*.

eTable 6. Survival Analysis and Marginal Structural Model for Developing Alzheimer Disease Dementia, Comparing the Cataract Surgery and No Surgery Groups

Risk ratios and 95% confidence intervals for the association of eye surgery as a time-varying exposure and subsequent Alzheimer disease as the outcome among people with cataract diagnosis are shown. All models use age as the time axis and adjust for education, self-reported White race, and past or current smoking, and are stratified by any *APOE* ε4 alleles and sex (to meet proportional hazards assumptions) and also age at first diagnosis of cataract (under 68, 68-71, 72-76, and 77 or older). These models are the same as those reported in **Table 2** except that these use probable or possible Alzheimer disease dementia as the outcome rather than all-cause dementia.

					**Time since surgery**[a]	
Models			**Surgery exposure (time-varying)**		**> 0 to 5 years**	**Over 5 years**
Model 1	Primary model		0.72 (0.61, 0.85)		0.65 (0.53, 0.80)	0.81 (0.66, 0.99)
Sensitivity analyses:						
1a	Omit 1994-6 enrollment cohort		0.50 (0.37, 0.68)		0.44 (0.31, 0.64)	0.62 (0.40, 0.97)
1b	Exclude surgery 2 years prior to censoring		0.60 (0.51, 0.70)		0.43 (0.34, 0.55)	0.73 (0.61, 0.88)
1c	Adjust for additional covariates		0.76 (0.64, 0.90)		0.69 (0.56, 0.84)	0.85 (0.69, 1.04)
1d	Consider only incident cataract cases		0.67 (0.53, 0.85)		0.63 (0.48, 0.84)	0.72 (0.53, 0.98)
1e	Incident cataract cases, controlling for CASI at time of cataract diagnosis		0.67 (0.53, 0.85)		0.64 (0.48, 0.85)	0.73 (0.54, 0.98)
1f	Adjust recent vs long-term threshold to 2-year window		--		0.59 (0.43, 0.80)	0.76 (0.64, 0.91)
1g	Adjust recent vs long-term threshold to 10-year window		--		0.72 (0.61, 0.85)	0.72 (0.53, 0.98)
Model 2	Marginal structural model with weights for surgery, death, and dropout to account for healthy patient bias		0.73 (0.60, 0.88)		0.75 (0.61, 0.92)	0.69 (0.53, 0.91)
Sensitivity analyses:						

© 2021 Lee CS et al. *JAMA Internal Medicine*.

2a	Marginal structural model with weights for surgery only		0.76 (0.63, 0.92)		0.77 (0.63, 0.94)	0.74 (0.56, 0.97)
2b	Adjust for additional covariates		0.74 (0.62, 0.90)		0.75 (0.61, 0.92)	0.73 (0.55, 0.96)
Model 3	Glaucoma surgery		1.25 (0.85, 1.86)		1.30 (0.77, 2.14)	1.19 (0.68, 2.09)

[a]A time-dependent covariate which is set at the first category (>0 to 5 years) for the first 5 years following surgery, and then to the second (over 5 years) after that.

Model 1 covered 23,554 person-years with 709 incident Alzheimer disease dementia cases. 432 cases occurred during the 15941 person-years before or without cataract surgery (0.027 per person-year), and 277 occurred during the 7603 person-years after surgery (0.036 per person-year).

Model 1a excludes the original Adult Changes in Thought (ACT) cohort recruited between 1994-1996 (resulting n=2868).

Model 1b ignored any cataract surgeries occurring within 2 years of Alzheimer disease diagnosis or censoring.

Model 1c is Model 1 additionally adjusted for diabetes, systolic blood pressure, hypertension, heart disease, cardiovascular disease, body mass index, self-rated health, Charlson comorbidity index (CCI), number of activity of daily living (ADL) and instrumental activity of daily living (IADL) limitations, at least 15 minutes of physical activity three times a week, performance-based physical function scores, Centers for Epidemiologic Studies Depression Scale (CESD) scores, and retirement status.

Model 1d includes only patients with first cataract diagnosis after ACT study entry (n=1556).

Model 1d adds CASI score at time of diagnosis to Model 1d.

Model 1f limits the recent cataract category to >0 to 2 years.

Model 1g expands the recent cataract category to >0 to 10 years.

Model 2 used stabilized time-varying weights in a marginal structural model to adjust for the probability of surgery, death, and dropout. (See Methods S2, Tables S2-4 for details).

Model 2a used stabilized time-varying weights in a marginal structural model adjusting only for the probability of surgery. (See Methods S2, Tables S2-4 for details).

Model 2b is Model 2 additionally controlled for diabetes, systolic blood pressure, hypertension, heart disease, cardiovascular disease, body mass index, self-rated health, CCI, number of ADL and IADL limitations, at least 15 minutes of physical activity three times a week, performance-based physical function scores, CESD scores, and retirement status.

Model 3 is a survival analysis with the same covariates and Alzheimer disease dementia outcome as in Model 1 but with the exposure of interest as history of glaucoma surgery instead of cataract surgery (n=728) and risk starting with the first glaucoma diagnosis. During 5029 person-years of follow-up, there were 184 incident Alzheimer disease dementia cases. 151 cases occurred during the 4497 person-years before or without glaucoma surgery (0.034 per person-year), and 33 occurred during the 533 person-years after surgery (0.062 per person-year).

© 2021 Lee CS et al. *JAMA Internal Medicine*.

eTable 7. Marginal Structural Models (MSM) for Alzheimer Disease (AD) Dementia With No Weights and With Un-Winsorized Weights

Risk ratios and 95% confidence intervals for the association of eye surgery and subsequent AD dementia among people with cataract diagnosis are shown. Age was the time axis, starting at baseline if the first cataract diagnosis was before baseline, or at incident cataract diagnosis. These MSM were adjusted for age of cataract diagnosis, sex, education, ≥1 *APOE* ε4 alleles, self-reported White race, and smoking history.

				Time since surgery[a]	
**AD Dementia**		**Surgery exposure (time-varying)**		**> 0 to 5 years**	**Over 5 years**
Model 1 from Table eTable 6:<br>Primary Cox model.		0.72 (0.61, 0.85)		0.65 (0.53, 0.80)	0.81 (0.66, 0.99)
Unweighted MSM:<br>Logit model to approximate a Cox model		0.75 (0.63, 0.88)		0.75 (0.62, 0.90)	0.74 (0.58, 0.95)
Model 2a from eTable 6:<br>MSM with surgery weights only		0.76 (0.63, 0.92)		0.77 (0.63, 0.94)	0.74 (0.56, 0.97)
MSM with weights for surgery, death, and attrition, not Winsorized		0.43 (0.25, 0.77)		0.48 (0.28, 0.83)	0.36 (0.19, 0.69)
Model 2 from eTable 6:<br>MSM with Winsorized weights for surgery, death, and attrition		0.73 (0.60, 0.88)		0.75 (0.61, 0.92)	0.69 (0.53, 0.91)

[a]A time-dependent covariate which is set at the first category (>0 to 5 years) for the first 5 years following surgery, and then to the second (over 5 years) after that.

© 2021 Lee CS et al. *JAMA Internal Medicine*.

eTable 8. Demographic and Health Variables at First Glaucoma Diagnosis by Later Surgery Status, Among Those Diagnosed With Glaucoma

	All (N=728)		Any surgery (N=105)		No surgery (N=623)		
	N or mean	% or SD	N or mean	% or SD	N or mean	% or SD	p-value[a]
Female	458	63%	69	66%	389	62%	0.52
Age at ACT study baseline	75.6	6.7	75.6	6.6	75.6	6.7	0.97
Age at first glaucoma diagnosis	76.3	8.4	74.8	7.6	76.6	8.5	0.03
Education (years)	14.6	3.1	14.0	3.1	14.6	3.1	0.09
Self-reported White race	651	89%	96	91%	555	89%	0.47
Any *APOE* ε4 alleles	183	25%	36	34%	147	24%	0.019
Past or current smoker	364	50%	51	49%	313	50%	0.73
Body Mass Index	26.9	4.8	27.1	4.9	26.8	4.7	0.62
Ever reported having diabetes	102	14%	12	11%	90	15%	0.39
Ever reported having hypertension	347	48%	42	40%	305	49%	0.08
Prevalent MI, angina, CABG, or angioplasty	132	18%	16	15%	116	19%	0.41
Ever reported stroke, TIA, or CEA	81	11%	10	10%	71	11%	0.58
CESD score	4.0	4.4	4.1	5.2	4.0	4.3	0.68
Systolic Blood Pressure	141.1	21.0	140.6	21.4	141.2	20.9	0.83
Poor or fair self-rated health	131	18%	13	12%	118	19%	0.11
Any ADL impairments	177	24%	29	28%	148	24%	0.40
Number of ADL impairments	0.4	0.8	0.4	0.8	0.4	0.8	0.41
At least 15 min of activity 3 x per week	503	69%	74	70%	429	69%	0.74

[a]Chi-square for dichotomous variables, Wilcoxon rank-sum for continuous variables. SD: standard deviation; ACT: Adult Changes in Thought; MI: myocardial infarction; CABG: coronary artery bypass grafting; TIA: transient ischemic attack; CESD: Center for Epidemiologic Studies Depression Scale; ADL: activity of daily living.

© 2021 Lee CS et al. *JAMA Internal Medicine*.

eReferences.

1. Kukull WA, Higdon R, Bowen JD, et al. Dementia and Alzheimer Disease Incidence. *Arch Neurol*. 2002;59(11):1737.

2. Larson EB, Wang L, Bowen JD, et al. Exercise is associated with reduced risk for incident dementia among persons 65 years of age and older. *Ann Intern Med*. 2006;144(2):73-81.

3. Crane PK, Gibbons LE, McCurry SM, et al. Importance of home study visit capacity in dementia studies. *Alzheimers Dement*. 2016;12(4):419-426.

4. Teng EL, Hasegawa K, Homma A, et al. The Cognitive Abilities Screening Instrument (CASI): a practical test for cross-cultural epidemiological studies of dementia. *Int Psychogeriatr*. 1994;6(1):45-58; discussion 62.

5. Crane PK, Trittschuh E, Mukherjee S, et al. Incidence of cognitively defined late-onset Alzheimer's dementia subgroups from a prospective cohort study. *Alzheimer's & Dementia*. 2017;13(12):1307-1316. doi:10.1016/j.jalz.2017.04.011

6. Spreen O, Spreen of PO, Strauss E, Strauss of PE. *A Compendium of Neuropsychological Tests: Administration, Norms, and Commentary*. Oxford University Press, USA; 1998.

7. Morris JC, Heyman A, Mohs RC, et al. The Consortium to Establish a Registry for Alzheimer's Disease (CERAD). Part I. Clinical and neuropsychological assessment of Alzheimer's disease. *Neurology*. 1989;39(9):1159-1165.

8. Mattis S. *Dementia Rating Scale Professional Manual*. Psychological Assessment Resources; 1988.

9. Wechsler D. *WMS-R: Wechsler Memory Scale--Revised : Manual*. Psychological Corporation; 1987.

10. Reitan RM, Wolfson D. *The Halstead-Reitan Neuropsychological Test Battery: Theory and Clinical Interpretation*. Vol 4. Reitan Neuropsychology; 1985.

11. Naj AC, Jun G, Beecham GW, et al. Common variants at MS4A4/MS4A6E, CD2AP, CD33 and EPHA1 are associated with late-onset Alzheimer's disease. *Nat Genet*. 2011;43(5):436-441.

12. American Psychiatric Association. *Diagnostic and Statistical Manual of Mental Disorders (4th Ed.)*. American Psychiatric Publishing, Inc.; 1994.

13. McKhann GM, Knopman DS, Chertkow H, et al. The diagnosis of dementia due to Alzheimer's disease: recommendations from the National Institute on Aging-Alzheimer's Association workgroups on diagnostic guidelines for Alzheimer's disease. *Alzheimers Dement*. 2011;7(3):263-269.

14. Blessed G, Tomlinson BE, Roth M. The association between quantitative measures of dementia and of senile change in the cerebral grey matter of elderly subjects. *Br J Psychiatry*. 1968;114(512):797-811.

15. Jorm AF, Jacomb PA. The Informant Questionnaire on Cognitive Decline in the Elderly (IQCODE): socio-demographic correlates, reliability, validity and some norms. *Psychological Medicine*. 1989;19(4):1015-1022. doi:10.1017/s0033291700005742

16. Robins JM, Greenland S, Hu F-C. Estimation of the Causal Effect of a Time-Varying Exposure on the Marginal Mean of a Repeated Binary Outcome. *J Am Stat Assoc*. 1999;94(447):687-700.

17. Cole SR, Hernán MA. Constructing Inverse Probability Weights for Marginal Structural Models. *American Journal of Epidemiology*. 2008;168(6):656-664.

18. Hernán MA, Brumback B, Robins JM. Marginal structural models to estimate the causal effect of zidovudine on the survival of HIV-positive men. *Epidemiology*. 2000;11(5):561-570.

© 2021 Lee CS et al. *JAMA Internal Medicine*.

19. Xu S, Ross C, Raebel MA, Shetterly S, Blanchette C, Smith D. Use of stabilized inverse propensity scores as weights to directly estimate relative risk and its confidence intervals. *Value Health*. 2010;13(2):273-277.

© 2021 Lee CS et al. *JAMA Internal Medicine*.
